# Prognostic and immunological role of sulfatide-related lncRNAs in hepatocellular carcinoma

**DOI:** 10.3389/fonc.2023.1091132

**Published:** 2023-02-01

**Authors:** Xing Feng Huang, Li Sheng Fu, Qian Qian Cai, Fei Fan

**Affiliations:** ^1^ Department of Biliary Tract Surgery, Shanghai Eastern Hepatobiliary Surgery Hospital, Shanghai, China; ^2^ Department of Biochemistry and Molecular Biology, School of Basic Medical Sciences, Fudan University, Key Lab of Glycoconjugate Research, Ministry of Public Health, Shanghai, China; ^3^ Shanghai Key Laboratory of Molecular Imaging, Shanghai University of Medicine and Health Sciences, Shanghai, China; ^4^ Department of The Second Ward of Special Treatment, Shanghai Eastern Hepatobiliary Surgery Hospital, Shanghai, China

**Keywords:** sulfatide, lncRNAs, HCC, prognosis, immune infiltration

## Abstract

**Background:**

Hepatocellular carcinoma (HCC) is the most common primary malignancy of the liver. Long non-coding RNAs (lncRNAs) play important roles in the occurrence and development of HCC through multiple pathways. Our previous study reported the specific molecular mechanism for sulfatide regulation of integrin αV expression and cell adhesion in HCC cells through lncRNA AY927503. Next, it is necessary to identify more sulfatide-related lncRNAs, explore their clinical signifcance, and determine new targeted treatment strategies.

**Methods:**

Microarrays were used to screen a complete set of lncRNAs with different expression profiles in sulfatide-treated cells. Sulfatide-related lncRNAs expression data and corresponding HCC patient survival information were obtained from the The Cancer Genome Atlas (TCGA) database, and the prognosis prediction model was constructed based on Cox regression analysis. Methylated RNA immunoprecipitation with next generation sequencing (MeRIP-seq) was used to detemine the effect of sulfatide on lncRNAs m6A modification. Tumor Immune Estimation Resource (TIMER) and Gene set nnrichment analysis (GSEA) were utilized to enrich the immune and functional pathways of sulfatide-related lncRNAs.

**Results:**

A total of 85 differentially expressed lncRNAs (|Fold Change (FC)|>2, *P*<0.05) were screened in sulfatide-treated HCC cells. As a result, 24 sulfatide-related lncRNAs were highly expressed in HCC tissues, six of which were associated with poor prognosis in HCC patients. Based on thses data, a sulfatide-related lncRNAs prognosis assessment model for HCC was constructed. According to this risk score analysis, the overall survival (OS) curve showed that the OS of high-risk patients was significantly lower than that of low-risk patients (P<0.05). Notably, the expression difference in sulfatide-related lncRNA NRSN2-AS1 may be related to sulfatide-induced RNA m6A methylation. In addition, the expression level of NRSN2-AS1 was significantly positively correlated with immune cell infiltration in HCC and participated in the peroxisome and Peroxisome proliferator-activated receptor (PPAR) signaling pathways.

**Conclusions:**

In conclusion, sulfatide-related lncRNAs might be promising prognostic and therapeutic targets for HCC.

## Background

Hepatocellular carcinoma (HCC) is the most common liver malignancy worldwide and is one of the top five deadliest cancers, with high morbidity and mortality rates (1). The level of sulfatide, a sulfoglycolipid, is usually elevated in HCC (2). and can protect hepatocytes from ischemia/reperfusion injury (3, 4). We previously reported that sulfatide enhances integrin aV (ITGAV) expression, leading to HCC metastasis (2, 5). Sulfatide is also abnormally expressed in ovarian carcinoma and renal cell carcinoma (6), and can be used as a specific biomarker for these tumors (7, 8). Moreover, direct inhibition of sulfatide biosynthesis by zoledronic acid can significantly inhibit the migration, invasion and lung metastasis of basal-like breast cancer cells (9). In our earlier study, lncRNA AY927503 was identified in sulfatide-treated HCC cells. It promoted HCC metastasis by inducing *ITGAV* transcriptional chromatin modification and was a potential molecular marker of HCC metastasis or poor prognosis (10). Long non-coding RNAs (lncRNAs) are RNA molecules consisting of more than 200 nucleotides with no or limited protein-coding potential, which affect tumor proliferation, migration and metastasis in the process of malignant tumor development (11). Therefore, further research on sulfatide-related lncRNAs will not only expand our understanding of the role of sulfatide in the occurrence and development of HCC, but may also provide potential prognostic biomarkers and individualized therapeutic targets for HCC. The present study sought to determine the role of the sulfatide-related lncRNAs in HCC.

## Methods

### Cell culture and treatment

SMMC-7721 cells were obtained from Cell Bank of Type Culture Collection of Shanghai Institute of Biochemistry & Cell Biology, Chinese Academy of Science. They were cultured in Dulbecco’s Modified Eagle’s Medium (Gibco, California, USA) supplemented with 10% fetal bovine serum (FBS) (Gibco). SMMC-7721 cells were identified by their morphological characteristics which were consistent with the establishment report (12). Cells were not contaminated by mycoplasma or infected with bacteria or fungi. All cells were cultured in a humidified incubator with 5% CO_2_ at 37°C. For the sulfatide treatment, cells were incubated at the initial density of 0.5×10^5^ cells/mL and treated with 2 μM galactocerebroside (Gal-Cer) or sulfatide (Sigma, St. Louis, MO, USA).

### Microarray expression profiling for lncRNA

Microarray profiling was conducted in the laboratory of Aksomics Inc. (Shanghai, China). The microarray was analyzed using the nrStar™ Functional LncRNA PCR chip software, version 1.0 (ArrayStar, Rockville, MD, USA). The hierarchical clustering analysis was carried out using a platform-independent software TBtools (version x64_1_09867) (13).

### Assessment of sulfatide-related lncRNAs expression in TCGA-LIHC

The TCGA Liver Cancer project (TCGA-LIHC) (N=423) data were downloaded from the UCSC Xena database (https://xenabrowser.net/) (14). Log2(x+0.001) transformation was used to standardize every gene expression profiles and noncoding genes were identified based on their Ensemble gene IDs.

### Survival prognosis analysis

A Cox proportional-hazards regression model was established to analyze the relationship between sulfatide-related lncRNA expression and overall survival (OS) in HCC. The patients were divided into two groups according to the best cutoff value for each sulfatide-related lncRNA, which calculated by the R package maxstat. The OS significance map in HCC was evaluated using the Kaplan-Meier plotter (http://kmplot.com/analysis/) (15).

### Prognostic risk score calculation

The least absolute shrinkage and selection operator (LASSO) regression analysis was conducted on the sulfatide-related lncRNAs. The LASSO regression algorithm was used for feature selection with 10-fold cross-validation. The R package glmnet was used for the analysis. For Kaplan-Meier curves, p-values and hazard ratios (HRs) with 95% confidence intervals (CIs) were generated using log-rank tests and univariate Cox proportional -hazards regression. Finally, six sulfatide-related lncRNAs were selected for incorporation into the risk score. The regression coefficient β for multivariate Cox regression model and lncRNA expression were used to construct the risk score formula as follows:

### Immune infiltration analysis

The Tumor Immune Estimation Resource (TIMER) database (http://timer.cistrome.org/) (16) analyzes immune cell infiltration in tumor tissues using high-throughput sequencing (RNA-Seq expression profiling) data (16, 17). The B cell, CD4+ T cells, CD8+ T cells, neutrophil, macrophage and dendritic cells infiltration score of HCC are evaluated by the Timer method of IOBR (version 0.99.9) (18), an R software package, based on the expression profile data of TCGA-LIHC. Spearman’s correlation coefficient between NRSN2-AS1 and immune cell infiltration score in HCC was calculated using corr.test function of R package psych (version 2.1.6) to determine the significantly correlated immune infiltration score.

### m6A-modified RNA immunoprecipitation sequencing

Total RNA samples were extracted, ragmented to 100bp, and immunoprecipitated using anti-m6A antibody (abcam). Then, eluted RNA and MeRIPed RNA were analyzed using deep sequencing with an Illumina Novaseq™ 6000 platform on the CLOUDSEQ Bio-tech Ltd (Shanghai, China) following the vendor’s recommended protocol (19)l.

### Biological signaling pathway analysis

Pathway enrichment analysis of Kyoto Encyclopedia of Genes and Genomes (KEGG) was performed using the Gene Set Enrichment Analysis (GSEA) database in TCGA-LIHC, which classified the data into high- and low- expression groups based on their *NRSN2-AS1* expression (20). Gene sets with |normalized enrichment score (NES)|>1, nominal p value (NOM *P)* <0.05, and false discovery rate (FDR) *q <*0.25 were considered to have significant enrichment.

### Statistical analysis

Differences in the expression of sulfatide-related lncRNAs between normal and tumor samples from each tumor were analyzed for significance using unpaired Wilcoxon rank sum and signed rank tests. Survival curves were statistically tested using the log rank test, where p-values and HRs with 95% CIs were represented *via* Kaplan-Meier plots. The significant correlations between sulfatide-related lncRNAs expression and immune cell infiltration scores in HCC were determined by analyzing Spearman’s correlation coefficients. Pearson correlation analysis was performed between the expression level of NRSN2-AS1_(ENSG00000225377) and gene set expression level of RNA m6A methylation-modifying enzyme. The level of significance was set at *P* < 0.05. The bioinformatics analysis platform Sangerbox, version 3.0 (http://vip.sangerbox.com/) (21), was used for processing of all the statistical analyses.

## Results

### Sulfatide induced differential expression of multiple lncRNAs in HCC cells

**Figure 1:**
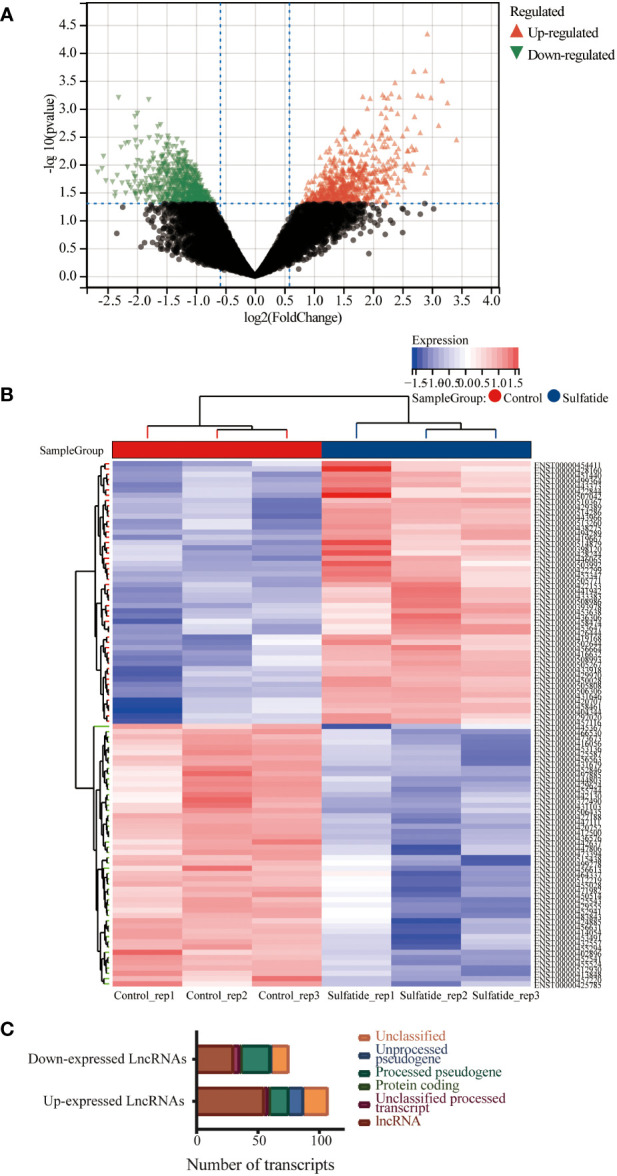
Multiple lncRNAs were differentially expressed in HCC cells after sulfatide treatment. **(A)** Volcano plot representing differentially expressed lncRNAs in HCC cells after sulfatide treatment. **(B)** Heat map showing differentially expressed lncRNAs in sulfatide-treated HCC cells and control cells. **(C)** Transcript classification analysis of differentially expressed lncRNAs.

The lncRNA profiles of sulfatide-treated HCC cells were compared to those of control cells using ArrayStar lncRNA microarray V2.0. This comparison identified 85 differentially expressed lncRNAs (DE-lncRNAs) based on their Ensemble IDs (|FC|>2, *P* < 0.05) (**Figures 1A, B**. **Supplementary Table 1**). These DE-lncRNAs were further classified by biotype, most of which were lncRNAs as processed transcripts, unclassified processed transcripts, processing/unprocessed pseudogenes, and small amounts of protein coding transcripts or unclassified transcripts (**Figure 1C**).

### Identification of differentially expressed sulfatide-related lncRNAs

**Figure 2:**
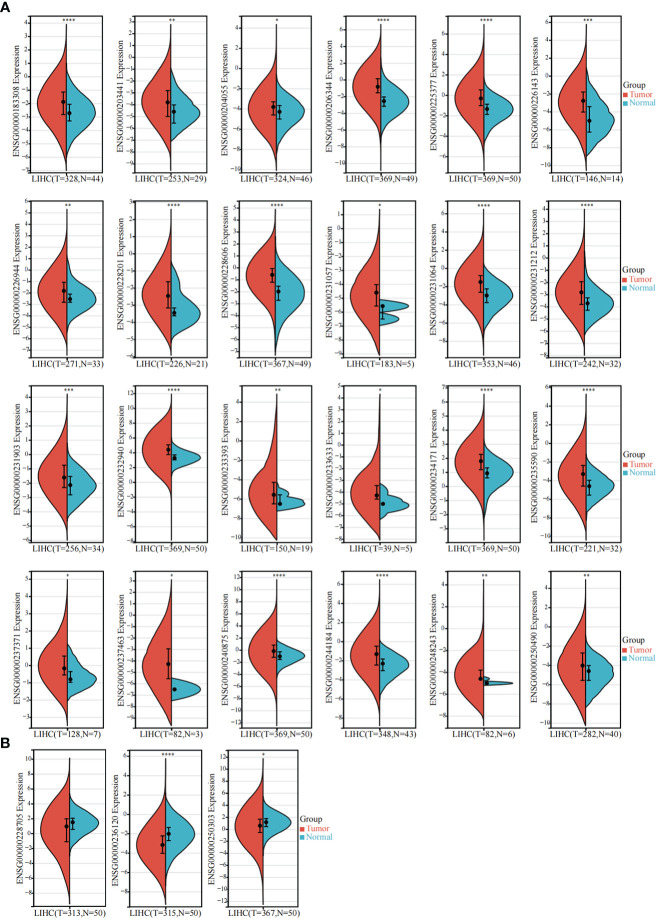
Differentially expressed sulfatide-related lncRNAs in TCGA-LIHC. **(A)** Screening of highly expressed sulfatide-related lncRNAs in TCGA-LIHC. **(B)** Sulfatide-related lncRNAs with low expression in TCGA-LIHC. *, p<0.05. **, p<0.01. ***, p<0.001. ****, p<0.0001.

The TCGA-LIHC dataset was used to detect the expression of these 85 sulfatide-related lncRNAs in HCC tissues, showing that 24 of them were highly expressed in HCC compared to normal liver tissues (**Figure 2A**). However, the expression of three sulfatide-related lncRNAs, AP002841.2 (ENST00000504610), RP11-733O18.1 (ENST00000422914) and RP5-885L7.10 (ENST00000412500) were lower in HCC tissues (**Figure 2B**).

### Prognostic value of sulfatide-related lncRNAs

**Figure 3:**
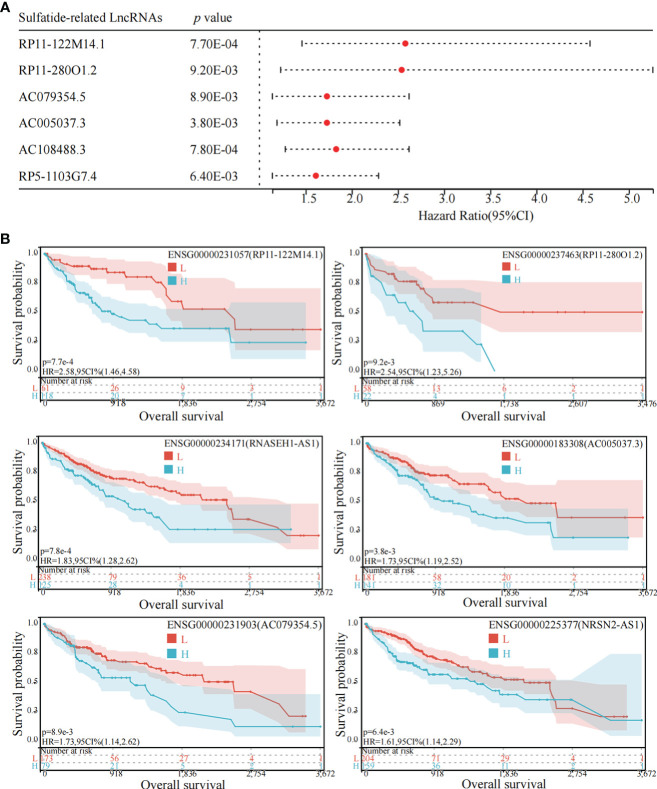
Identification of sulfatide-related lncRNAs with prognostic value in HCC patients. **(A)** Univariate Cox regression analysis of six differentially expressed sulfatide-related lncRNAs and risk scores in HCC samples. **(B)** Kaplan-Meier analytical evaluation of prognostic values of six differentially expressed sulfatide-related lncRNAs.

Among the above mentioned 27 sulfatide-related lncRNAs, six lncRNAs were ultimately identified to be related to prognosis (**Figure 3A**), including RP11-122M14.1 (ENST00000415202), RP11-280O1.2 (also known as LRRC52-AS1; ENST00000438275), AC079354.5 (ENST00000447111), AC005037.3 (ENST00000413848), AC108488.3 (also known as RNASEH1-AS1; ENST00000438436) and RP5-1103G7.4 (also known as NRSN2-AS1; ENST00000442637). Kaplan–Meier survival analysis was utilized to evaluate the significance of lncRNA expression in patient prognosis (**Figure 3B**). High levels of these sulfatide-related lncRNAs were all correlated with poor prognosis in patients with HCC (**Figure 3**).

### Construction of prognostic signature based on sulfatide-related lncRNAs

**Figure 4:**
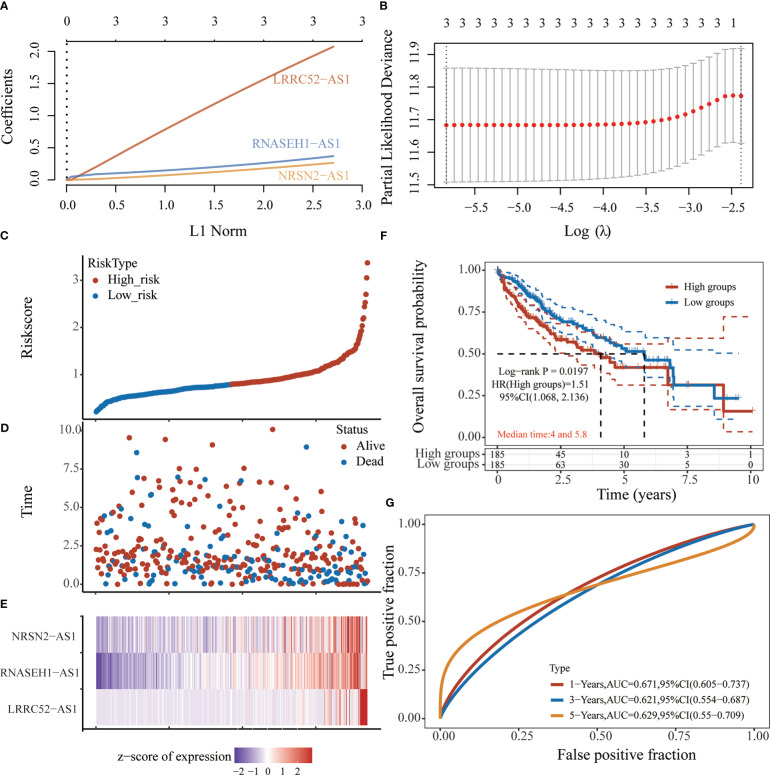
Prognostic risk score characteristics of sulfatide-related lncRNAs in HCC. **(A)**, **(B)** LASSO Cox regression with 10-fold cross-validation of the prognostic value of three sulfatide-related lncRNAs, including LRRC52-AS1, RNASEH1-AS1, and NRSN2-AS1. C, **(D)** Risk curve **(C)** and scatter plot **(D)** for the risk score and survival status of each HCC case. Blue and red dots in **(D)** represent death and survival, respectively. **(E)** Heat map showing the expression profiles of three sulfatide-related lncRNAs in the high-risk and low-risk group. **(F)** Kaplan-Meier prognostic prediction analyses of risk score model at 10-year timepoint. **(G)** Time-dependent receiver operating characteristic curves for the prognostic prediction of risk score models at one-, three-, and five-year time points.

Based on the expression of six sulfatide-related lncRNAs and multivariate Cox regression coefficient, the prognosis risk score for sulfatide-related lncRNAs was calculated using the following formula: riskscore (lambda.min=0.0029) = (2.0727) × LRRC52-AS1 + (0.3691) × RNASEH1-AS1 + (0.2646) × NRSN2-AS1 (**Figures 4A, B** **)**. Subsequently, an X-tile diagram was used to generate the optimal cutoff point for the risk score. The TCGA-LIHC patients were divided into high- and low-risk groups based on this cutoff risk score value. A prognostic curve and a scatter plot were used to indicate the risk score and survival status of each HCC patient (**Figures 4C, D** **)**. In addition, the heat map of the expression profiles for candidate lncRNAs demonstrated that they were all highly expressed in the high-risk group (**Figure 4E**). Kaplan-Meier analysis validated that the TCGA-LIHC patients in the high-risk group showed a significantly worse survival than those in the low-risk group at the 10-year time point (**Figure 4F**). Furthermore, the time-dependent receiver operating characteristic (ROC) analyses showed that the area under curve (AUC) for the risk score model was 0.671 at the one-year time point, 0.621 at the three-year time point, and 0.629 at the five-year time point (**Figure 4G**). Taken together, these findings represented the three sulfatide-related lncRNAs as the prognostic signature for HCC patients.

### NRSN2-AS1 expression was associated with RNA m6A methylation

**Figure 5:**
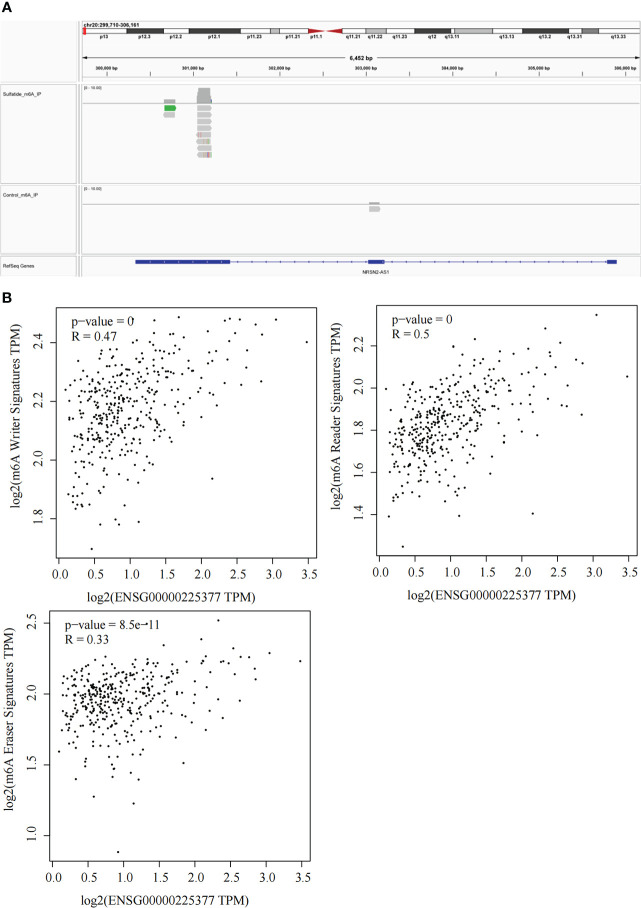
Sulfatide affects NRSN2-AS1 expression by regulating the RNA m6A methylation modification. **(A)** Abundances of m6A in NRSN2-AS1 determined by MeRIP-seq. **(B)** Pearson correlation of NRSN2-AS1 expression and m6A writer, reader, and eraser signature expressions.

Our previous study reported that sulfatide does not only affect the binding of METTL3 to METTL14 and WTAP by acetylating the METTL3 protein (19), but also inhibits the YTHDF2 expression in HCC cells (22). Next, we investigated whether RNA m6A methylation modification affected sulfatide-related lncRNA expression. The MeRIP-seq experiments were performed, in order to clarify the role of RNA m6A methylation modification. Their results showed that the abundance of m6A in NRSN2-AS1, one of sulfatide-related lncRNAs, was significantly increased in sulfatide-treated HCC cells. This suggested that m6A modification was related to the regulation of NRSN2-AS1 expression (**Figure 5A**). Furthermore, the relationship between NRSN2-AS1 expression level and a series of m6A-binding proteins (23) was analyzed in TCGA-LIHC samples. The results showed that the expression levels of NRSN2-AS1 were positively correlated with the expression of m6A writer and reader signatures (**Figure 5B**). In summary, the expression of NRSN2-AS1 in HCC was related to the changes in RNA m6A methylation induced by sulfatide.

### Role of NRSN2-AS1 in HCC immune microenvironment characterization

**Figure 6:**
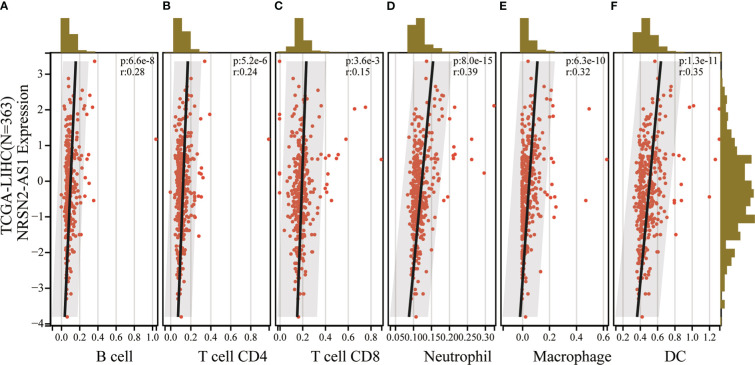
Correlation of NRSN2-AS1 in tumor immune microenvironment characterization. **(A-F)**. Correlation between NRSN2-AS1 expression and immune infiltration level of B cells **(A)**, CD4+ T cells **(B)**, CD8+ T cells **(C)**, neutrophils **(D)**, macrophages **(E)**, and dendritic cells **(F)** in HCC.

An increasing number of studies have demonstrated that sulfatide is involved in tumor immunity, where the HIF-1-galactose-3-o-mercaptotransferase 1-sulfide axis enhanced immune escape of renal clear cell carcinoma by increasing tumor cell-platelet binding (24). In addition, a subpopulation of type II Natural killer T cells (NKT cells) characterized by their response to autoglycolipid sulfides was shown to induce a major immunomodulatory mechanism that controls inflammation in anticancer immunity (25). To further examine whether the sulfatide-related lncRNA NRSN2-AS1 can act as an immune indicator, a correlation analysis of NRSN2-AS1 expression with immune infiltration was performed. TIMER data showed that high NRSN2-AS1 expression was significantly associated with six types of immune cells (B cells, CD4+ T cells, CD8+ T cells, macrophages, neutrophils and dendritic cells) in HCC (**Figure 6**). This result pointed out that NRSN2-AS1 may serve as an indicator in tumor immune microenvironment (TIME) characterization in HCC.

### Functional enrichment analysis of NRSN2-AS1 in HCC

**Figure 7:**
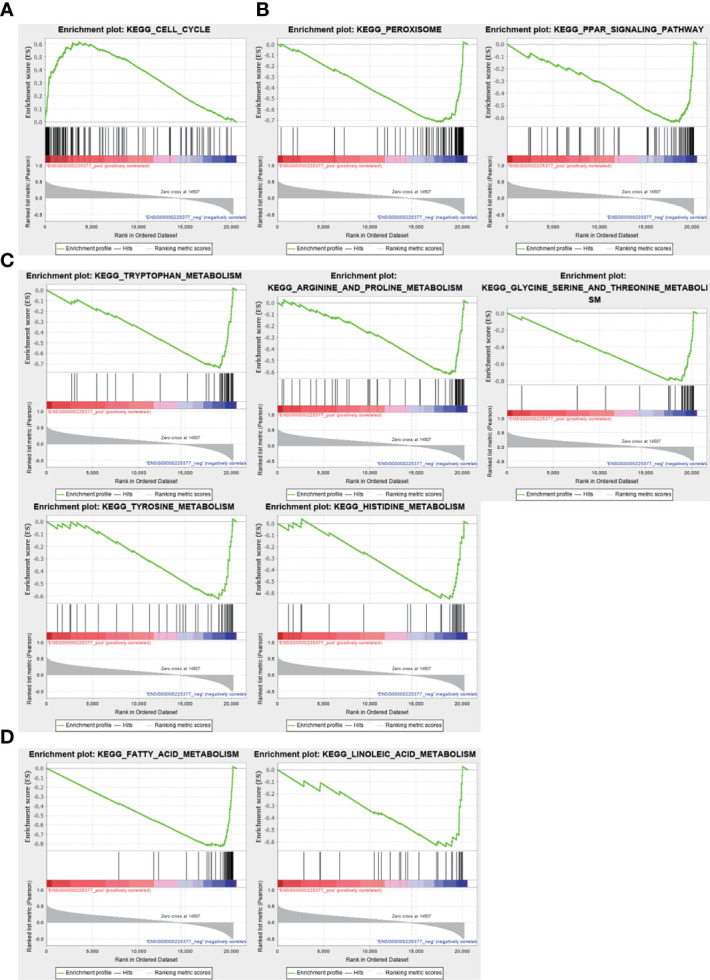
Gene set enrichment analysis for NRSN2-AS1. **(A)**. Significantly enriched pathways in patients with high NRSN2-AS1 expression. **(B-D)**. Significantly enriched pathways in patients with low NRSN2-AS1 expression.

To investigate the biological functions and pathways associated with the sulfatide-related lncRNA NRSN2-AS1, the TCGA-LIHC samples were divided into high- and low-expression groups based on their NRSN2-AS1 expression. GSEA was used to evaluate the enrichment of KEGG pathways. The pathways associated with high NRSN2-AS1 expression were enriched in the Cell Cycle pathway (**Figure 7A**). The pathways associated with low expression of NRSN2-AS1 were enriched in peroxisome and perxisome proliferator-activated receptor (PPAR) signaling pathways related to immune response (26, 27), as well as a variety of amino acid (tryptophan, arginine, proline, glycine, serine, threonine, tyrosine, and histidine) and lipid (fatty acid and linoleic acid) metabolic pathways (**Figures 7B-D**).

## Discussion

HCC is the most frequently occurring type of primary liver cancer, and its pathogenesis involves a complex transcriptional regulation disorder (28–30) and energy metabolism abnormality (31–33). Therefore, identifying reliable and effective biomarkers for HCC prognosis is of great importance. Glycosylsphingolipids (GSLs) are important components of cell membranes and act as signaling molecules in cellular processes. Similar to GSLs, sulfatide (glycosphingolipid sulfate) is also composed of lipid and sugar components, and its precursor galactosylceramide connects the sulfate ester group to the carbohydrate component in the endoplasmic reticulum (ER) (34). Elevated expression of sulfatide has been found in many human cancer cell lines and tissues, and can be used as a biomarker of some cancers (4, 35, 36). Abundant sulfatide on the surface of cancer cells is a natural ligand of P-selectin ligand that helps to promote tumor metastasis (37, 38). Many lncRNAs are abnormally expressed in various cancers, including HCC, and play a key role in tumorigenesis (39). We previously reported the abundant expression of sulfatide in HCC (5), and investigated the specific molecular mechanism for sulfatide regulation of integrin αV expression and cell adhesion in HCC cells *via* lncRNA AY927503 (10, 22, 40). However, the effect of sulfatide on the expression levels of other lncRNAs in HCC cells and the role of these DE-lncRNAs in prognosis and immunotherapy evaluation require further study.

The present study screened 85 DE-lncRNAs (|FC|>2, *P*<0.05) in sulfatide-treated HCC cells based on their Ensemble IDs. Tthe TCGA-LIHC database 27 sulfatide-related lncRNAs that were differentially expressed in HCC and adjacent tissues, of which 24 were highly expressed in HCC tissues. RP11-122M14.1, RP11-280O1.2, AC079354.5, AC005037.3, AC108488.3 and RP5-1103G7.4 are six sulfatide-related lncRNAs with abnormally high expression that were significantly associated with poor prognosis in HCC patients. When selecting specific variables to build the prognosis evaluation model, overfitting often occurs if too many variables are present (41). Regularization is an important method to solve the overfitting problem (42). LASSO regression constructs a penalty function and adds L1 regularization after the loss function to obtain a more accurate model with fewer variables (43). After the LASSO regression analysis of six lncRNAs, only three were found to be related to the patient prognosis. Based on the risk score results and sulfatide-related lncRNA construction, the OS for high-risk patients was significantly lower than that for low-risk patients (*P*<0.05).

Sulfatide had been demonstrated to be one of several natural ligands for type II CD1d-restricted NKT cells, which can regulate tumor immunity (36, 44, 45). More and more studies have also found the potential effect of lncRNAs on immune cells infiltration in TIME. For example, lncRNA MIAT is distributed in HCC. It is enriched in FOXP3+CD4+T, PDCD1+CD8+, and GZMK+CD8+T cells, affects the immune microenvironment of HCC by regulating the expression of target genes JAK2, SLC6A6, KCND1, MEIS3, and RIN1, and participates in the immune escape process in HCC (46). The lncRNA MIAT also mediates HCC immune response by targeting the miR-411-5p/STAT3/PD-L1 axis (47). Therefore, we speculated that sulfated-related lncRNAs may also be involved in regulating the HCC TIME. Based on the TIMER database, we confirmed that the high expression of sulfatide-related lncRNA NRSN2-AS1 was significantly related to the infiltration of immune cells, such as macrophages, dendritic cells, neutrophils, B cells, CD4+T cells, and CD8+T cells in HCC. As a newly identified lncRNA, NRSN2-AS1 has not been well studied in cancer. The latest research found that NRSN2-AS1 is significantly overexpressed in ovarian cancer, plays a tumor-promoting role as the sponge of miR-744-5p, and regulates the Wnt/β-catenin signaling pathway *via* the miR-744-5p/PRKX axis (48). It was also found that SOX2 promotes NRSN2-AS1 transcription in esophageal squamous cell carcinoma (ESCC), and that NRSN2-AS1 promotes its progression by regulating the ubiquitin degradation of PGK1 (49). However, the role and mechanism of NRSN2-AS1 in tumor immunity remain unknown.

The GSEA results suggested that the pathways related to the low expression of NRSN2-AS1 are mainly enriched in the peroxisome and PPAR signaling pathways. Peroxisome proliferator-activated receptors (PPARs) belong to the nuclear hormone receptor family. They are divided into α, β, and γ subtypes, and participate in the metabolism of various energy substances and tumor immunity. PPARα was found to respond to the fatty acids delivered by tumor-derived exosomes (TDEs), resulting in excess lipid droplet biogenesis and enhanced fatty acid oxidation (FAO), culminating in a metabolic shift toward mitochondrial oxidative phosphorylation, which drives tumor-infiltrating DCs (TIDCs) immune dysfunction (50). It was reported that CD36 is selectively upregulated in intrautumoral Treg cells as a central metabolic modulator activates PPARβ signaling to regulate mitochondrial adaptation, and programs Treg cells to adapt to lactic acid-enriched TME (51). PPARγ is selectively expressed in group 2 innate lymphoid cells (ILC2s) supported the IL-33-dependent tumor promoting effect (27). The PPARγ-dependent upregulation of FAO also mediates the pro-tumor (also known as M2-like) polarization of tumor-associated macrophages (TAMs) (52). Tumor infiltrating T cells have also been found to have a progressive loss of PPAR-gamma coactivator 1α (PGC1α), which programs mitochondrial biogenesis, induced by chronic Akt signaling. This results in continuous loss of mitochondrial function and quality of tumor-specific T cells (53). These results provide a possible direction for further research on the role and mechanism of NRSN2-AS1 in HCC tumor immunity.

## Conclusions

In this study, we described the influence of sulfatide on lncRNA expression in HCC cells and found that these sulfatide-related lncRNAs serve as a good prognostic marker for HCC patients. In addition, we showed that NRSN2-AS1 may be an indicator of TIME characterization in HCC. These results help to improve the understanding of the comprehensive characteristics and role of sulfatide in the development and progression of HCC and will help to optimize immunotherapy regimens.

## Data availability statement

The datasets presented in this study can be found in online repositories. The names of the repository/repositories and accession number(s) can be found below: https://www.ncbi.nlm.nih.gov/geo/, GSE151111.

## Author contributionss

The concept of the project was proposed by FF and QC. XH and QC collected the data from the databases and performed data analysis. The MeRIP-seq cell samples were cultured and prepared by LF. XH wrote the manuscript, and QC and FF contributed to editing and participated in manuscript-related discussions. All authors contributed to the article and approved the submitted version.
