# Supplementary material for: Prognostic and immunological role of sulfatide-related lncRNAs in hepatocellular carcinoma
Source: Front Oncol. 2023 Feb 1;13:1091132. doi: 10.3389/fonc.2023.1091132 (PMC9929346; doi:10.3389/fonc.2023.1091132)
Supplement: Supplementary file 1 [file Table_1.docx]

Supplemental Table 1. List of differential expression LncRNAs (Sulfatide treated SMMC-7721 vs. Gal-Cer treated SMMC-7721, (|Fold Change|>2, *P* < 0.05 ))

| Seqname | logFC | P.Value | adj.P.Val |
| --- | --- | --- | --- |
| ENST00000508993 | 3.264434967 | 0.000777013 | 0.366663314 |
| ENST00000458461 | 3.114366 | 0.001975933 | 0.366663314 |
| ENST00000451440 | 2.983176767 | 0.000627642 | 0.357673325 |
| ENST00000443373 | 2.835866733 | 0.000591935 | 0.357673325 |
| ENST00000419168 | 2.7059568 | 0.001377103 | 0.366663314 |
| ENST00000442482 | 2.686028867 | 0.005408942 | 0.366663314 |
| ENST00000440609 | 2.6801432 | 0.009709625 | 0.366663314 |
| ENST00000474149 | 2.5979443 | 0.005664861 | 0.366663314 |
| ENST00000447384 | 2.5852312 | 0.005590338 | 0.366663314 |
| ENST00000454411 | 2.578991167 | 0.002256851 | 0.366663314 |
| ENST00000502644 | 2.505050533 | 0.003038233 | 0.366663314 |
| ENST00000433918 | 2.446479933 | 0.000607087 | 0.357673325 |
| ENST00000504610 | 2.433681267 | 0.005125818 | 0.366663314 |
| ENST00000415801 | 2.423699 | 0.007168774 | 0.366663314 |
| ENST00000505267 | 2.418658267 | 0.001214286 | 0.366663314 |
| ENST00000419576 | 2.392776333 | 0.005964999 | 0.366663314 |
| ENST00000422799 | 2.3744408 | 0.000338722 | 0.357673325 |
| ENST00000428160 | 2.366836633 | 0.004131727 | 0.366663314 |
| ENST00000417447 | 2.302494867 | 0.007886184 | 0.366663314 |
| ENST00000448001 | 2.2533805 | 0.008910154 | 0.366663314 |
| ENST00000453347 | 2.2522001 | 0.00063689 | 0.357673325 |
| ENST00000448491 | 2.2501218 | 0.004680625 | 0.366663314 |
| ENST00000420707 | 2.2484172 | 0.003355783 | 0.366663314 |
| ENST00000422844 | 2.238775267 | 0.002280967 | 0.366663314 |
| ENST00000511758 | 2.1985997 | 0.004789381 | 0.366663314 |
| ENST00000457116 | 2.194915633 | 0.003817022 | 0.366663314 |
| ENST00000419667 | 2.1642023 | 0.000559953 | 0.357673325 |
| ENST00000514879 | 2.148799033 | 0.002672305 | 0.366663314 |
| ENST00000427859 | 2.058837967 | 0.007481687 | 0.366663314 |
| ENST00000416861 | 2.035079033 | 0.005176492 | 0.366663314 |
| ENST00000422473 | 2.0036996 | 0.004469965 | 0.366663314 |
| ENST00000438659 | 1.996369 | 0.007379305 | 0.366663314 |
| ENST00000436306 | 1.989398433 | 0.00307855 | 0.366663314 |
| ENST00000415202 | 1.9279895 | 0.005444018 | 0.366663314 |
| ENST00000443966 | 1.905110367 | 0.000985999 | 0.366663314 |
| ENST00000414654 | 1.876179 | 0.009721554 | 0.366663314 |
| ENST00000431646 | 1.8281311 | 0.000602358 | 0.357673325 |
| ENST00000422914 | 1.726763 | 0.005945979 | 0.366663314 |
| ENST00000438275 | 1.6734673 | 0.003081237 | 0.366663314 |
| ENST00000433085 | 1.6603401 | 0.00904923 | 0.366663314 |
| ENST00000420389 | 1.611496333 | 0.008548778 | 0.366663314 |
| ENST00000422153 | 1.5399155 | 0.003460776 | 0.366663314 |
| ENST00000429389 | 1.5049929 | 0.002262927 | 0.366663314 |
| ENST00000451707 | 1.4938117 | 0.00905533 | 0.366663314 |
| ENST00000508986 | 1.476130533 | 0.002603348 | 0.366663314 |
| ENST00000453968 | 1.4709128 | 0.006143556 | 0.366663314 |
| ENST00000423246 | 1.444878233 | 0.00573344 | 0.366663314 |
| ENST00000458001 | 1.433951667 | 0.008350631 | 0.366663314 |
| ENST00000441942 | 1.427435267 | 0.004264007 | 0.366663314 |
| ENST00000356006 | 1.345745233 | 0.007456748 | 0.366663314 |
| ENST00000514010 | 1.344992 | 0.008161035 | 0.366663314 |
| ENST00000444079 | 1.323432467 | 0.009647252 | 0.366663314 |
| ENST00000455038 | 1.267605533 | 0.007956805 | 0.366663314 |
| ENST00000416046 | 1.2638178 | 0.005929332 | 0.366663314 |
| ENST00000510416 | 1.1064438 | 0.007322005 | 0.366663314 |
| ENST00000506435 | -1.1900439 | 0.007810014 | 0.366663314 |
| ENST00000447111 | -1.206146833 | 0.005732459 | 0.366663314 |
| ENST00000366259 | -1.2284389 | 0.008668554 | 0.366663314 |
| ENST00000456563 | -1.229342333 | 0.005796883 | 0.366663314 |
| ENST00000455744 | -1.2742164 | 0.00659466 | 0.366663314 |
| ENST00000455294 | -1.2827388 | 0.006330254 | 0.366663314 |
| ENST00000413848 | -1.3026554 | 0.006380491 | 0.366663314 |
| ENST00000473352 | -1.3187105 | 0.009125488 | 0.366663314 |
| ENST00000425587 | -1.3208799 | 0.007060395 | 0.366663314 |
| ENST00000412500 | -1.323241867 | 0.003536955 | 0.366663314 |
| ENST00000455524 | -1.338365 | 0.004824748 | 0.366663314 |
| ENST00000438436 | -1.3515559 | 0.008694864 | 0.366663314 |
| ENST00000453136 | -1.3990654 | 0.004790824 | 0.366663314 |
| ENST00000442130 | -1.4057232 | 0.007033814 | 0.366663314 |
| ENST00000442637 | -1.4659867 | 0.004492015 | 0.366663314 |
| ENST00000450514 | -1.4699003 | 0.006753367 | 0.366663314 |
| ENST00000431103 | -1.477717933 | 0.00312568 | 0.366663314 |
| ENST00000497885 | -1.498971767 | 0.00461876 | 0.366663314 |
| ENST00000414054 | -1.499931133 | 0.004346653 | 0.366663314 |
| ENST00000456613 | -1.521612433 | 0.008067723 | 0.366663314 |
| ENST00000424675 | -1.532730267 | 0.009787292 | 0.366663314 |
| ENST00000512219 | -1.5558021 | 0.006796828 | 0.366663314 |
| ENST00000372490 | -1.573468533 | 0.007301247 | 0.366663314 |
| ENST00000483843 | -1.5779082 | 0.007192849 | 0.366663314 |
| ENST00000455028 | -1.585952167 | 0.007669207 | 0.366663314 |
| ENST00000452846 | -1.645620567 | 0.002113721 | 0.366663314 |
| ENST00000438428 | -1.765396533 | 0.008879082 | 0.366663314 |
| ENST00000503218 | -1.799312433 | 0.009428317 | 0.366663314 |
| ENST00000427188 | -1.800730233 | 0.000695438 | 0.361434616 |
| ENST00000456631 | -2.016370733 | 0.002190429 | 0.366663314 |
